# Effect of Structure and Composition of Non-Stoichiometry Magnesium Aluminate Spinel on Water Adsorption

**DOI:** 10.3390/ma13143195

**Published:** 2020-07-17

**Authors:** Yuval Mordekovitz, Yael Shoval, Natali Froumin, Shmuel Hayun

**Affiliations:** 1 Department of Materials Engineering, Ben-Gurion University of the Negev, P.O. Box 653, Beer-Sheva 84105, Israel; yuvalmor@post.bgu.ac.il (Y.M.); yaelsh5@gmail.com (Y.S.); nfrum@bgu.ac.il (N.F.); 2 Ilsa katz Institute for Nanoscience and Technology, Ben-Gurion University of the Negev, P.O. Box 653, Beer-Sheva 84105, Israel

**Keywords:** water adsorption, defect structure, reducibility, magnesium aluminate spinel

## Abstract

MgAl_2_O_4_ is used in humidity sensing and measurement, and as a catalyst or catalyst support in a wide variety of applications. For such applications, a detailed understanding of the surface properties and defect structure of the spinel, and, in particular, of the gas interactions at the spinel surface is essential. However, to the best of our knowledge, very limited experimental data regarding this subject is currently available. In this work, four spinel samples with an Al_2_O_3_ to MgO ratio (*n*) between 0.95 and 2.45 were synthesized and analyzed using X-ray photoelectron spectroscopy and water adsorption micro-calorimetry. The results showed that the spinel composition and its consequent defect structure do indeed have a distinct effect on the spinel-water vapor surface interactions. The adsorption behavior at the spinel-water interface showed changes that resulted from alterations in types and energetic diversity of adsorption sites, affecting both H_2_O uptake and overall energetics. Furthermore, changes in composition following appropriate thermal treatment were shown to have a major effect on the reducibility of the spinel which enabled increased water uptake at the surface. In addition to non-stoichiometry, the impact of intrinsic anti-site defects on the water-surface interaction was investigated. These defects were also shown to promote water uptake. Our results show that by composition modification and subsequent thermal treatments, the defect structure can be modified and controlled, allowing for the possibility of specifically designed spinels for water interactions.

## 1. Introduction

Magnesium aluminate spinel (MgAl_2_O_4_, MAS) has been shown to be useful for humidity sensing and measurement applications, and as a catalyst or catalyst support for various organic reactions [1,2,3,4,5,6,7]. For all these applications, a detailed understanding of the surface properties of the spinel, and specifically the nature of spinel gas-surface interactions, is paramount. Nevertheless, to date, very limited data is available regarding water-surface interactions on MAS and their relation to spinel defect structure, and the information that is available is largely based on theoretical calculations [8]. MAS is the only stable intermediate phase in the Al_2_O_3_-MgO system, and at elevated temperatures (i.e., over 1300 °C), this system exhibits a large non-stoichiometric range [9], which can also exist at lower temperatures as a metastable nanomaterial [10,11,12].

The spinel structure has a general formula of AB_2_O_4_, with the lattice comprising an almost perfect, close-packed cubic arrangement containing 32 oxygen anions. In this arrangement, the A and B cations are situated inside the tetrahedral and octahedral interstitials, respectively. In MAS, eight Mg^2+^ cations are located in the tetrahedral sites, and sixteen Al^3+^ cations occupy the octahedral sites [10,13,14,15]. The defect structure of spinel is comprised of intrinsic (i.e., Frenkel, Schottky and anti-site) defects and extrinsic (i.e., non-stoichiometric, dopant or impurity) defects. It has been established that the dominating intrinsic defect in MgAl_2_O_4_ spinel is the anti-site defect (AKA inversion), in which two cations switch places. Specifically, an Mg^2+^ occupies the Al^3+^ octahedral site and vice versa [16,17,18,19,20].

The inversion level (i.e., the number of tetrahedral sites occupied by Al^3+^ cations) is controlled by three major factors. The first is related to the thermal history of the material, reflecting the “*intrinsic*” defect concentration. The other two are due to extrinsic parameters, the first of which is MAS stoichiometry (i.e., the. “*stoichiometric*” factor), and the second is residual disorder resulting from thermal effects and stresses in the material synthesis process (i.e., the “*residual*” inversion).

The *intrinsic* component can be calculated using a thermodynamic model, such as that developed by O’Neill and Navrotsky [21], using experimental parameters [10]. The *stoichiometric* component can be quantified from the total defect concentration [13]. The residual inversion, resulting from the synthesis process, cannot be assessed accurately, but this type of inversion defect can be manipulated and subsequently reordered using external fields [10,13].

For MAS, the shift away from idealized stoichiometry can be considered to be due to the dissolution of MgO or Al_2_O_3_ in the spinel matrix. Departing from the stoichiometric ratio in either the Al_2_O_3_- or MgO-rich direction, results in different structural defects. Spinel crystals with excess Al_2_O_3_ are characterized by $Al_{Mg,}^{•},$ which can be charge-compensated by $V_{Mg}^{\prime \prime }$, $V_{Al}^{\prime \prime \prime }$ or their combination [16,22,23,24,25], as demonstrated in the following equations:

(1) $$4\cdot {\mathrm{Al}}_{2}O_{3}\to 5\cdot {\mathrm{Al}}_{\mathrm{Al}}^{\times }+12\cdot O_{O}^{\times }+3\cdot {\mathrm{Al}}_{\mathrm{Mg}}^{•}+V_{\mathrm{Al}}^{\prime \prime \prime }$$

(2) $$4\cdot {\mathrm{Al}}_{2}O_{3}\to 6\cdot {\mathrm{Al}}_{\mathrm{Al}}^{\times }+12\cdot O_{O}^{\times }+2\cdot {\mathrm{Al}}_{\mathrm{Mg}}^{•}+V_{\mathrm{Mg}}^{\prime \prime }$$

(3) $$12\cdot {\mathrm{Al}}_{2}O_{3}\to 16\cdot {\mathrm{Al}}_{\mathrm{Al}}^{\times }+36\cdot O_{O}^{\times }+8\cdot {\mathrm{Al}}_{\mathrm{Mg}}^{•}+V_{\mathrm{Mg}}^{\prime \prime }+2\cdot V_{\mathrm{Al}}^{\prime \prime \prime }$$

Alternatively, spinel crystals with excess MgO incorporate $Mg_{Al}^{\prime }$ defects. In this case, the preferred charge compensation would be in the form of $V_{O}^{••}$ [16,22,23,24,25], as described by:

(4) $$3MgO\to 2Mg_{Al}^{\prime }+Mg_{Mg}^{X}+3O_{O}^{X}+V_{O}^{••}$$

Using the Brouwer diagram, the defect types in MAS can be described in terms of the Al_2_O_3_ content [23] according to the following guidelines:

(5) $$\frac{Mg_{Al}^{\prime }/V_{O}^{••}}{Low\;{\mathrm{Al}}_{2}O_{3}}\leftarrow \frac{{\mathrm{Al}}_{\mathrm{Mg}}^{•}/Mg_{\mathrm{Al}}^{\prime }}{MgAl_{2}O_{4}}\to \frac{{\mathrm{Al}}_{\mathrm{Mg}}^{•}/V_{\mathrm{Al}}^{\prime \prime \prime }}{Moderate\;{\mathrm{Al}}_{2}O_{3}}\to \frac{{\mathrm{Al}}_{\mathrm{Mg}}^{•}/V_{\mathrm{Mg}}^{\prime \prime }}{High\;{\mathrm{Al}}_{2}O_{3}}$$

The type and quantity of defects both in the bulk and on the surface of the material changes as a function of stoichiometry. Changes in defect structure can be used for tuning the properties of a material [14,26,27], including its surface properties [2]. Surface properties are also affected by the environmental state in which the material is maintained. For example, ambient, clean, reduced, oxidized or humid environments all have different effects on the surface state.

To the best of our knowledge, no experimental data regarding the effects of non-stoichiometry on surface-water interactions in the MgO*•n*Al_2_O_3_ system have been published, although some theoretical work has been performed. This paper, therefore, aims to study the effect of the surface composition on the interactions between a non-stoichiometric MgO*•n*Al_2_O_3_ spinel system and water vapor.

## 2. Materials and Methods

### 2.1. Materials

Nano-sized MgO*•n*Al_2_O_3_ powders with 0.95 < *n* < 2.45 were synthesized by the solution combustion method [28]. This entailed the mixing of appropriate amounts of magnesium and aluminum nitrate, Mg(NO_3_)_2_·6H_2_O 96% metal basis, Al(NO_3_)_3_·9H_2_O (96% metal basis, Fluka Analytical, Sigma Aldrich, St. Louis, MO, USA) in 200 mL of deionized water. To this solution, 30 g of citric acid (ACS reagent ≥99.5%) and 6 mL of ethylene glycol (anhydrous, 99.8%, Sigma Aldrich, St. Louis, MO, USA) were added. The solution was dried on a hot plate at 120 °C under agitation by magnetic stirring until high-viscosity foam-like colloids were formed. The foams were crushed using a mortar and pestle to a fine light brown powder, which served as precursors for the appropriate spinel. The precursors were calcined in air at 850 °C for 72 h to obtain fine white powders.

**Figure 1:**
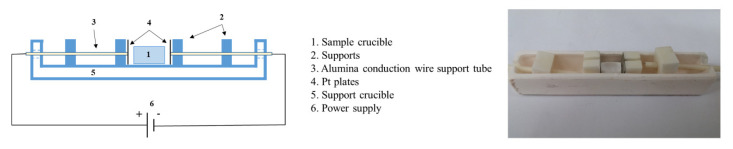
Crucible and cell used for the electric field heat treatments.

To study the effects of disorder on the adsorption process, some of the samples were heat-treated in the presence of an electric field, in order to reduce “residual inversion” defects. The samples were heated in air to 800 °C, and maintained at this temperature for 30 min. The heating rate was 10 °C/min, and the furnace was naturally cooled. An electric field of 200 V/cm was applied at all treatment stages. Figure 1 shows the crucible and capacitor setup used for the treatment. As can be seen, there was no contact between the powder and the electrodes, and there was no flow of current.

### 2.2. Characterization

X-ray diffraction (XRD) patterns of the samples were recorded using a Rigaku RINT 2100 (Tokyo, Japan) diffractometer with CuKα radiation. The operating parameters were 40 kV and 40 mA, with a 2θ step size of 0.02°. Si (NIST SRM 640c) served as internal standard for cell parameter determination. Crystallite sizes were refined from diffraction peak broadening using a whole profile fitting procedure, as implemented in the Jade software package (version 6.11, 2010, Materials Data Inc., Livermore, CA, USA).

Sample composition was determined by atomic absorption spectroscopy (AAS) using a Varian SpectrAA 240FS (currently Agilent Technologies, Santa-Clara, CA, USA).

Surface area was measured using the Brunauer–Emmett–Teller (BET) theory [29] using a Micrometrics ASAP 2020 (Micrometrics, Norcross, GA, USA) instrument. Fifteen-point adsorption isotherms of nitrogen were collected in the P/P_0_ relative pressure range 0.05–0.30, where P_0_ is the saturation pressure at −196 °C. Prior to analysis, each sample was degassed under vacuum at 700 °C for 4 h.

X-ray photoelectron spectroscopy (XPS) data was collected using an X-ray photoelectron spectrometer ESCALAB 250 (Thermo Fisher Scientific, Waltham, MA, USA) ultrahigh vacuum (10^−9^ bar) apparatus with an Al*K*^α^ X-ray source and a monochromator. The X-ray beam spot size was 500 μm, and survey spectra were recorded with pass energy (PE) of 150 eV. High-energy resolution spectra were recorded using 20 eV PE. To correct for charging effects, all spectra were calibrated relative to a carbon C1s peak positioned at 284.8 eV. Processing of the XPS results was carried out using the Thermo Scientific AVANTAGE program. For accurate surface characterization by XPS, a glove box was mounted on the XPS enter lock chamber to avoid adsorption of any species from the air on the samples. To ensure that all the samples were investigated under the same experimental conditions, all samples were equilibrated for 12 h in the entry lock chamber of the XPS prior to making the measurements.

IR spectra were recorded at room temperature using a Nicolet 6700 (Thermo Scientific, Madison, WI, USA) FT-IR spectrometer with a KBr-DTGS detector in the range spanning 400–4000  cm^−^^1^. Mixtures containing 100 mg KBr and 1 mg spinel were compressed at 1 ton to generate thin plates. For each material, 64 scans of the spectrum were recorded and averaged. The spectrometer settings were at aperture of 150 and spectral resolution of 4 cm^−1^. Peak positions and intensities were determined by OPUS software (Billerica, MA, USA) using the second derivative and standard methods. The averaged spectrum was used to calculate the inversion parameters of the samples, employing the method of Erukhimovitch et al. [10], which uses the intensity ratios of the γ_1_ and γ_5_ modes (FTIR peaks located at ~690 and ~830 cm^−1^).

### 2.3. Water Adsorption Calorimetry

The heat of adsorption of the water–spinel surface interactions was measured using a custom-made apparatus, composed of a volumetric sorption system (ASAP2020, Micromeritics, Norcross, GA, USA) and a differential scanning calorimeter (Sensys Calvet, Setaram, Lion, France) [30]. The instrumental design and its operation have been discussed elsewhere. Here, approximately 100 mg of sample powder was put inside the sample tube, providing a total surface area of 1.0–5.6 m^2^. The tube is than placed inside the calorimeter, it is also connected to the sorption system via a conceive tube. Prior to measurement, a degas procedure was performed. One was cooled to 25 °C at the end of the degassing process, whereas the second was exposed to an oxygen atmosphere prior to cooling down (i.e., oxidized/clean). All the samples were exposed to controlled, and incremental H_2_O vapor doses until the partial pressure reached P/P_0_ = 0.3. The incremental dose was set to provide 1 µmol of H_2_O vapor per m^2^ of sample surface. The heat of adsorption (ΔH_ads_) for each dose was measured. The measurements were repeated 3–4 times for each sample to ensure reproducibility. A baseline run was performed to eliminate environmental and instrumental contributions to the signal.

## 3. Results and Discussion

This study focused on the effects of surface composition and state on water–surface interactions for four different MgO*•n*Al_2_O_3_ spinel powders. The samples investigated comprised a series of nano-sized (10–15 nm) metastable spinels, with composition (*n*) ranging between 0.95 and 2.45 (Table 1). Their lattice parameters, crystallite sizes and surface areas are summarized in Table 1. The lattice parameter increased inversely with the n ratio, in keeping with literature results [31,32]. The surface areas measured by the BET method differed significantly from those calculated theoretically from XRD crystallite size (assuming spherical approximation), indicating that the samples had undergone extensive sintering during the final stages of their synthesis.

**Figure 2:**
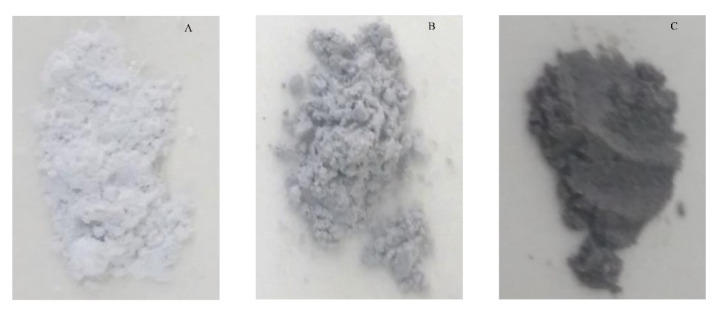
Different sample colors after degassing procedure showing different reduction level vs n: (**A**) n = 1.07; (**B**) n = 1.15; (**C**) n = 2.45.

Four types of surface states/conditions were addressed in this work: for simplicity, they will be referred to as “as synthesized” (AS), reduced (RD), clean (CL) and hydrated (HD). The AS condition refers to a sample after calcination. The RD condition refers to samples after the degassing procedure (Figure 2). It can be seen that the powders lost their original white color and became greyish-dark after the degassing procedure, with the samples that were richer in Al_2_O_3_ becoming markedly darker in the RD state, indicating that they were more easily reduced. Oxidation of the RD samples by exposure to 1 atm of oxygen at 700 °C and cooling to room temperature in a 1 atm oxygen environment resulted in the restoration of the original white color of the samples. It can be concluded that the dark color of RD samples was a result of the formation of oxygen vacancies during the degassing stage, which was reversed in the oxidization step by “refilling” the oxygen vacancies formed by the initial degas, while simultaneously keeping the surface clean. The reoxidized samples were designated CL. Finally, either RD or CL samples were exposed to H_2_O vapor in the water adsorption experiments to generate the HD states.

### 3.1. Surface State Analysis

**Figure 3:**
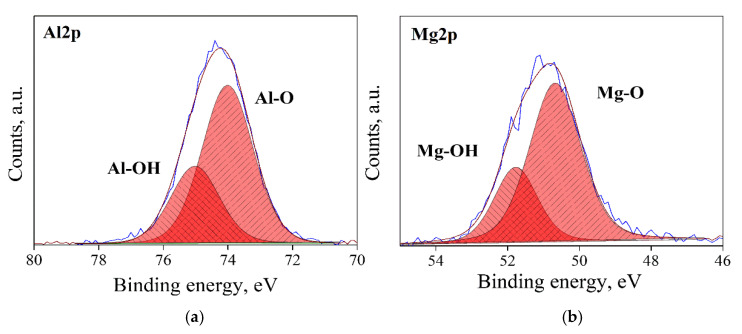
XPS spectra, of an AS, n = 1.07 sample: Al2p de-convoluted to Al–O and Al–OH peaks (**a**), and Mg2p, de-convoluted to Mg–O and Mg–OH peaks (**b**).

**Figure 4:**
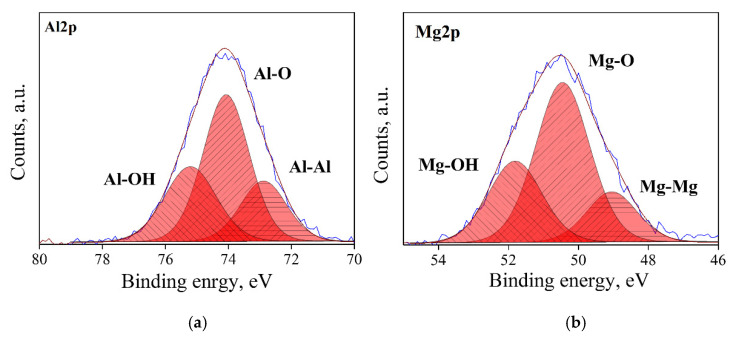
XPS spectra of an RD, n = 1.07 sample, after degassing and oxidation: Al2p de-convoluted to Al–O, Al–OH and Al–Al peaks (**a**), and Mg2p, de-convoluted to Mg–O, Mg–OH and Mg–Mg peaks (**b**).

**Figure 5:**
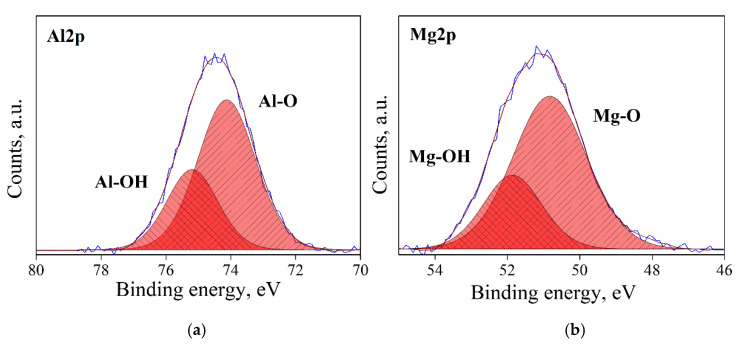
XPS spectra, of a CL, n = 1.07 sample, after degassing and oxidation: Al2p de-convoluted to Al–O and Al–OH peaks (**a**): and Mg2p deconvoluted to Mg–O and Mg–OH peaks (**b**).

**Figure 6:**
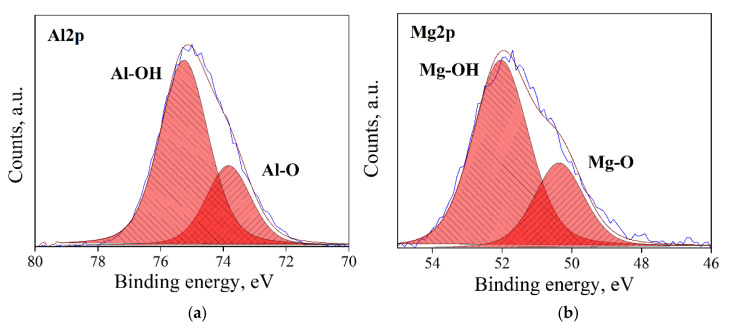
XPS spectra of an HD, n = 1.07 sample after degassing, oxidation, and exposure to water vapor: Al2p de-convoluted to Al–O and Al–OH peaks (**a**), and Mg2p, de-convoluted to Mg–O and Mg–OH peaks (**b**).

Typical XPS spectra in the Al2p and Mg2p regions for the four types of surface state (AS, RD, CL and HD) of MgO*•1.07*Al_2_O_3_ are shown Figure 3, Figure 4, Figure 5 and Figure 6. Three types of bonds, specifically M–M, M–O and M–OH bonds (M = Al, Mg), were considered for each spectrum. In the Al2p spectra, these are assigned at ~73.0, 74.1 and 75.2 eV, respectively [33,34,35]. In the Mg2p spectra, their assignments are ~49.3, 50.9 and 51.8, respectively [36]. The dominant bond in AS samples (Figure 3) is the M–O bond, with less prominent, though not negligible, M–OH bonds. The RD samples (Figure 4) displayed different spectra due to the prolonged degassing procedure. Here, the amount of the hydroxyl surface species was reduced, and the presence of M–M bonds was detected. These M–M Bonds were formed due to oxygen deficiencies in the structure, as reflected in the color changes of the powders (Figure 2). In the CL samples (Figure 5), the M–M bonds disappeared and were replaced by M–O species, with the quantity of M–OH bonds being lower than in the AS samples. As expected, the HD samples exhibited the highest quantity of M–OH bonds, and no M–M bonds were identified (Figure 6). This behavior was found to be typical for all the samples in this study. The data for all samples are given in the Supplementary Materials in Tables S1 and S2.

### 3.2. Water Adsorption Measurments

**Figure 7:**
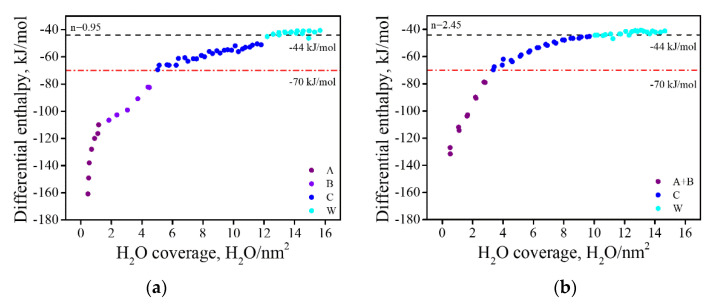
Differential enthalpies of adsorption as a function of water coverage on CL samples: an MgO-rich sample, n = 0.95 (**a**), and an Al_2_O_3_-rich sample, n = 2.45 (**b**). The blue line signifies the transition between strongly bonded and weakly bonded water, and the red line emphasizes enthalpy of −70 kJ/mol, where the site type of strongly bonded water is altered.

Heat of adsorption measurements were conducted for the reduced (RD) and fully oxidized (CL) samples. Typical heat of adsorption isotherms for MgO- and Al_2_O_3_-rich spinel samples are presented in Figure 7. It should be emphasized that in this calorimetric study, water molecules adsorbed with an enthalpy greater than −44 kJ/mol relative to vapor are referred to as “strongly bound”, while those adsorbed with the enthalpy of condensation of liquid water (−44 kJ/mol) are considered “weakly bound”. Furthermore, we should stress that such assignments are based solely on calorimetric data and do not reflect any structural studies of water adsorbed on the surface [37,38].

The heat of adsorption isotherms can be divided into segments, according to the change in their slope. Each segment of the isotherm may be considered as reflecting a different type of adsorption site, or site group [39,40]. The MgO-rich spinel displayed a four-step behavior (Figure 7a). In the first step, marked in purple (type A), a sharp decrease in the enthalpy of adsorption was seen, up to H_2_O coverage of ~2 molecules/nm^2^. From there, a second step was observed up to ~5 molecules/nm^2^, marked in violet (type B), in which the slope changed direction, and the decrease in ΔH_ads_ was moderated. At the transition point between the second and third steps ΔH_ads_ reached a value of ≈−70 kJ/mol. From this point of the curve, the blue section (type C), the ΔH_ads_ slowly decayed until the measured enthalpy corresponded to that of weakly bonded water. After reaching −44 kJ/mol (the cyan section of the curve, type D), the enthalpy fell to lower values (absolute) of about −39 kJ/mol. In general, the MgO-rich samples showed similar behavior to that of pure MgO, where surface hydroxides (i.e., Mg(OH)_2_) are formed [41].

In the case of the Al_2_O_3_-rich samples, the first two steps observed for the MgO-rich samples were combined into a single step (up to ~4 molecules/nm^2^, marked in purple, types A + B). After this point, a more moderate slope was identified, up to −44 kJ/mol (Figure 7b, blue colored, type C), with no further decreases.

**Figure 8:**
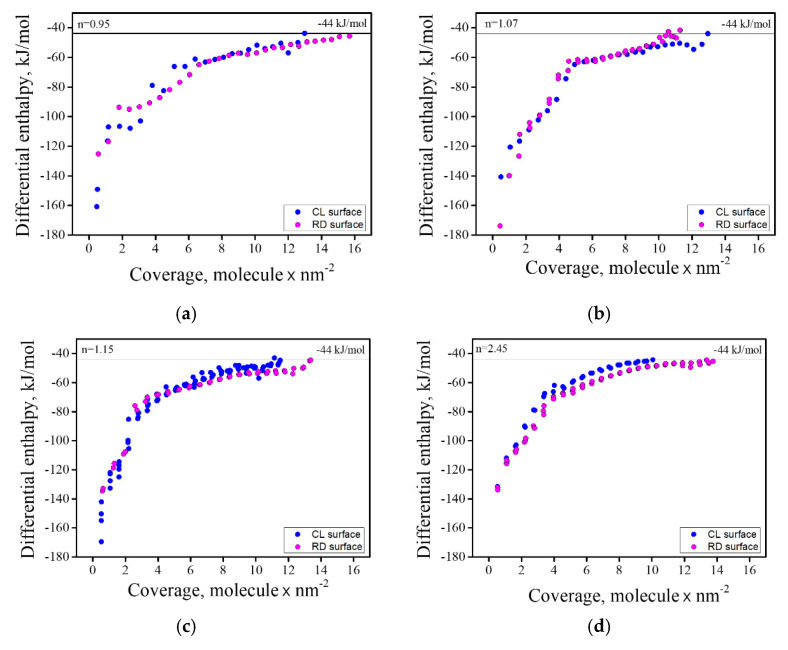
Heat of adsorption isotherms for all samples for RD and CL surfaces: (**a**) n = 0.95; (**b**) n = 1.07; (**c**) n = 1.15; (**d**) n = 2.45.

The heat of the adsorption isotherms for all samples, in their CL and RD states, can be seen in Figure 8. These isotherms are a depiction of the gas adsorption amount (Figures S1 and S2) obtained in each dose with their corresponding energetic value. Table 2 summarizes the integral heat of adsorption of strongly bond water and the water coverage for all samples, as well as the amounts of Mg and Al hydroxides formed on the surface, deduced from the XPS analysis. The columns in Table 2 under the heading “Hydroxides” present differences in the surface hydroxide compositions for the initial RD and CL states and after their hydration. The measured values are in good agreement with the water coverage, except for the n = 0.95 sample. We believe that XPS measurements do not accurately account for the coverage in this sample, possibly due to the presence of surface contamination by adventitious species on the CL/RD samples. Such species are thought to readily adsorb due to the defective nature of MgO rich spinel [16,22,23,24,25].

The results showed a clear relation between the Al_2_O_3_ concentration and the water adsorption in the CL samples, in terms of both water uptake and energetics. In general, as the Al_2_O_3_ concentration persisted in the reduced samples (RD), with the exception of the n = 1.07 sample. The possible origins for this apparent anomaly will be discussed below. Notably, each sample in the RD state accumulated more adsorbed water than its CL analogue (excluding n = 1.07), as its surface defect structure was altered. It is possible that some of the adsorbed water may act as an oxidizing agent, but we believe that this role is limited due to the relatively low temperature of the adsorption process.

Water uptake in the RD state was dependent on Al_2_O_3_ concentration, but this dependency is not as simple as was observed for the CL samples, even if the n = 1.07 sample is excluded. This, and the apparently anomalous behavior of the n = 1.07 sample, are attributed to the differences in the level of reduction as was seen by the changes in sample coloring. Based on the color differences of the Al_2_O_3_-rich samples (Figure 2), we concluded that the level of reduction increased with Al_2_O_3_ concentration, but the extent of reduction was not quantitatively determined in this work. The effects of reduction in enhancing the extent of adsorption were evident in the increased (and similar) extent of water uptake for the n = 1.15 and n = 2.45 samples. However, the integral enthalpy of adsorption of the RD samples was lowered, relative to their CL counterparts, as the defect structure was progressively altered. These alterations, in turn, resulted in changes in the site population and its energetic diversity, the source of which lies in the newly induced material defect structure.

In spinel, water molecules are adsorbed in the vicinity of the metal ions, specifically ${\mathrm{Al}}_{\mathrm{Al}}^{x},{\mathrm{Mg}}_{\mathrm{Mg}}^{x},{\mathrm{Al}}_{\mathrm{Mg}}^{•},\mathrm{and}\;{\mathrm{Mg}}_{\mathrm{Al}}^{\prime }$ [42]. As Al_2_O_3_ is added in excess, the Al^3+^ cations progressively occupy tetrahedral sites, substituting Mg^2+^ and disturbing the charge neutrality. To maintain charge neutrality, cation vacancies are formed, some of which are on the surface of the spinel structure [16,22,23,24,25]. Consequently, the quantity of surface cations is diminished and hence also the quantity of available adsorption sites which leads to lower quantities of adsorbed water.

An excess of Al_2_O_3_ also influences the proximity of the metal cations to the surface, as it affects which of the material planes have a higher tendency to be exposed. Cai et al. [43] calculated the surface stability of exposed spinel surfaces and concluded that in Al_2_O_3_-rich spinel 111_O_2_(Al) plane tends to be exposed. In this plane, oxygen molecules are slightly elevated over the Al^3+^ cations, thus decreasing the energy of interaction at the surface. As the material becomes poorer in Al_2_O_3_, the 100_Al(O_2_) plane is exposed [43]. This plane has a higher surface Gibbs free energy, thereby allowing for interactions at the surface that are more energetic.

It is important to note that the n = 0.95 samples are MgO-rich, and thus, the factors contributing to the defect structure are different. Here, oxygen vacancies compensate for excess Mg, which essentially allows for better exposure of the metal cations. Moreover, MgO is highly hygroscopic, forming a surface hydroxide phase that enables additional, more energetic water uptake [41]. In the RD state, oxygen vacancies were formed, their numbers growing with Al_2_O_3_ concentration, with consequent changes in the surface defect chemistry and electronic structure. In the RD samples, a decrease in the integral enthalpy of adsorption was registered, relative to their CL counterparts. To determine the origins of this decrease, a closer analysis of the energetic diversity of the adsorption sites is required.

**Figure 9:**
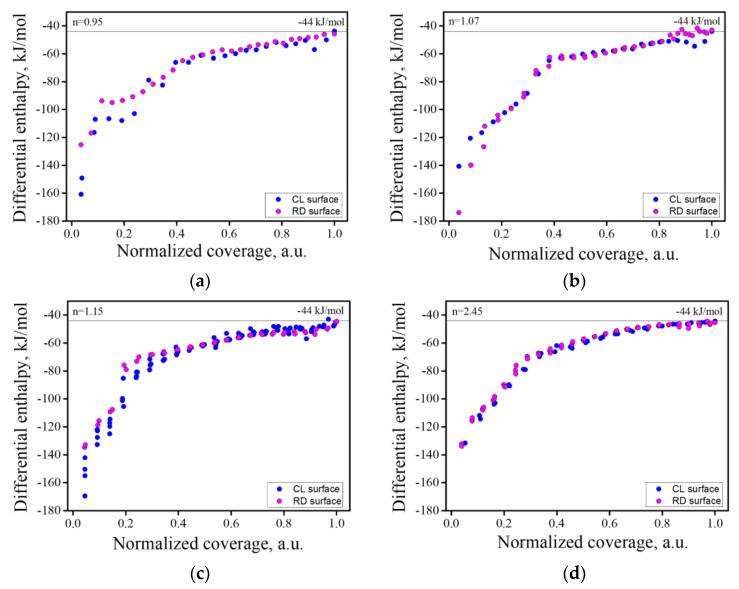
Heat of adsorption isotherms for all CL and RD samples. The coverage is normalized to the full coverage of strongly adsorbed water for each sample. (**a**) n = 0.95; (**b**) n = 1.07; (**c**) n = 1.15; (**d**) n = 2.45.

Figure 9 shows normalized heat of adsorption isotherms for CL and RD samples. In these isotherms, the *x*-axis was normalized to the amount adsorbed at full coverage for each sample. This observation allows us to consider the energetic distribution of the sites. In samples n = 0.95 and 1.15, the isotherms for the RD and CL materials are almost overlapped, with the exception of the very first few sites at low relative coverage (type A), which were more energetic for the CL samples than for their RD counterparts. An inverse relationship was obtained for the n = 1.07 sample. These first few (low relative coverage) sites are very energetic and make a considerable contribution to the overall energetics. The new adsorption sites that were added after reduction are of type C, which suggests water was unable to re-oxidize the surface. Finally, the CL and RD isotherms for the n = 2.45 sample were in almost perfect alignment, suggesting that the energetic diversity of the adsorption sites was maintained regardless of the reduction process undergone by this material. Accordingly, the integral enthalpy of adsorption for this sample in the two states was similar.

At this point, it is important to address the anomalous behavior exhibited observed for the sample n = 1.07. This material has near stoichiometric composition, and, as is evident from Figure 2, was barely reduced by the degassing procedure. Nonetheless, some changes in the defect structure did occur. Jia et al. [42] showed computationally that for stoichiometric ZnGa_2_O_4_ spinel, such reduction-related defects do not always enhance water adsorption [42]. We assume that a similar explanation is appropriate here because of the proximity of the n = 1.07 sample to stoichiometry. This implies that in order for the reduction process to enhance the surface reactivity, the composition should not be near stoichiometric.

### 3.3. Effect of Anti-Site Defects

**Figure 10:**
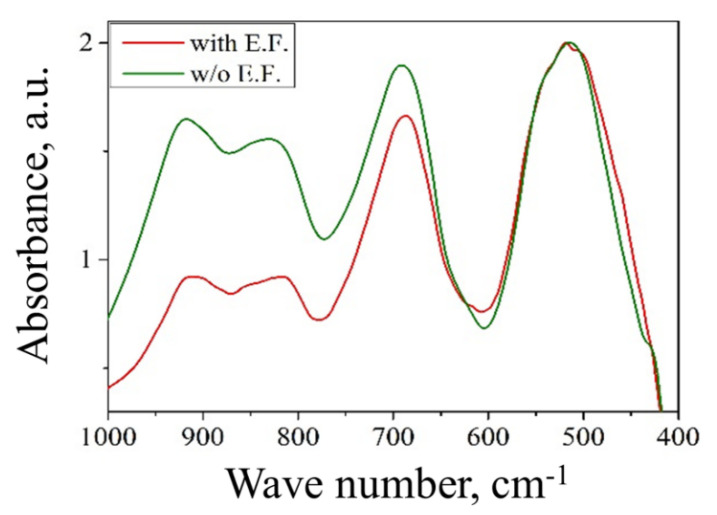
FTIR spectra of MgO•2.45Al_2_O_3_ before and after application of an electric field.

As discussed above, the spinel system is subject to extrinsic and intrinsic defects. The former, which can exert a considerable effect on water-surface interactions can, however, be influenced by controlling the composition of the material. The spinel system also presents intrinsic, anti-site defects that are not controllable or that are controllable only to a certain extent and require specific study and understanding. Thus, to assess the effects of anti-site defects on adsorption behavior, a MgO*•2.45*Al_2_O_3_ (*n* = 2.45) sample was heat-treated in the presence of a constant electric field (EF). FTIR spectra in the 400–1000 cm^−1^ range of the sample before and after the heat treatment are presented in Figure 10. The aim of the electric field treatment was essentially to rearrange the defects caused by the *residual* inversion without otherwise affecting the material. The thermal treatment was performed at 800 °C, a temperature lower than the calcination temperature of the sample (850 °C) so that any changes in the material as result of the heat treatment in the presence of the electric field can be attributed solely to the effect of the EF. The function of heating (to 800 °C) is to provide sufficient thermal energy to assist cation rearrangement, driven by the applied electric field. As a result of this treatment, highly disordered samples (*I* = 0.44) with *n* ratio of 2.45 underwent significant reordering (*I* = 0.33), as is qualitatively demonstrated in the FTIR spectrum by the decrease in the intensity of the γ_5_ mode (~830 cm^−1^) (Figure 10) [10].

**Figure 11:**
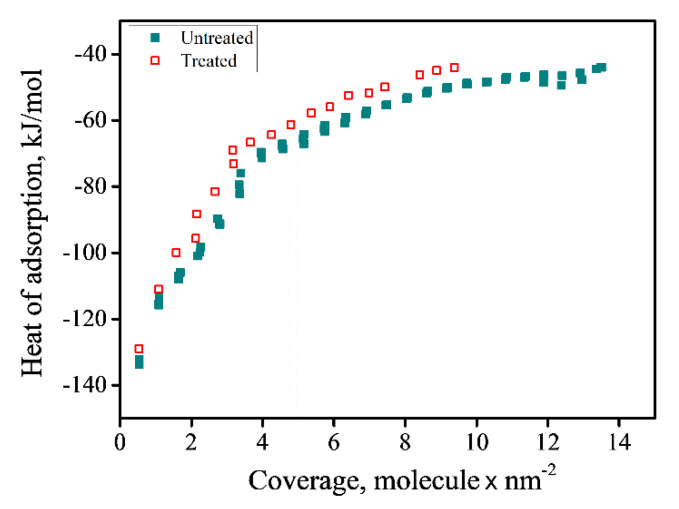
Differential enthalpy of adsorption as a function of water coverage of MgO•2.45Al_2_O_3_ samples before and after heat treatment in an electric field.

After the heat treatment and subsequent inversion parameter (i) decrease, water adsorption of this re-ordered sample was measured in the same way as for all the preceding materials. The adsorption enthalpy isotherms of the two spinel samples, before and after heat treatment with the electrical field are shown in Figure 11. From the data listed in Table 3, it is apparent that following reordering, the enthalpy of adsorption decreased, as did the coverage. The extent of formation of hydroxide bonds was determined using XPS (Table 3), and the results are in agreement with the results of the heat of adsorption experiments. Heat treatment together with the application of an electrical field led to significantly less hydroxides being formed, with the change in Al–OH bonding being more marked than that for Mg–OH. These experimental findings can be explained by the existence of excess charge when the Al^+3^ cations are located in the tetrahedral sites.

## 4. Conclusions

The relationship between the composition of spinels and their general properties is undeniable, and has been widely demonstrated in the literature. Surface behavior and water–surface interactions are no exception. The composition dictates, in part, the defect structure of a material. This, in turn, controls the surface behavior. Ultimately, these aspects govern the water–surface interaction.

We have shown that changes in stoichiometry alter the water adsorption behavior in our system. In general, an increase in the Al_2_O_3_ concentration lowers both water uptake and the energy of water adsorption. Changes in the Al_2_O_3_ concentration influence the defect structure of the material, thereby changing the adsorption site population and its energetic diversity. Furthermore, the material composition affects the reducibility of the material, and thus, its ability to host more defects. These defects promote water uptake while lowering the adsorption enthalpy.

In addition to the effects of non-stoichiometry, the effects of intrinsic defects in spinel should be considered when dealing with water–surface interactions. A spinel having a lower inversion parameter (i), i.e., a material with fewer anti-site defects, was shown to adsorb fewer strongly bonded water molecules and to present lower enthalpies of adsorption, indicating that the Al cation is more active when it occupies a tetrahedral site in the spinel structure.

## Figures and Tables

**Table 1:** As synthesized characteristics: lattice parameter, crystallite size, surface and interface areas.

n	(Mg_x_Al_y_O_4_)		Lattice Parameter, Å	Crystallite Size, nm	Surface Area, m^2^/g	
	(x) Mg	(y) Al			XRD	BET
0.95	1.04	1.97	8.089(2)	14.0 ± 0.2	117.7 ± 1.6	32.1 ± 0.2
1.07	0.95	2.03	8.078(2)	13.3 ± 0.2	123.9 ± 1.7	37.6 ± 0.2
1.15	0.72	2.18	8.065(6)	10.2 ± 0.3	161.6 ± 4.9	56.4 ± 0.2
2.45	0.48	2.35	7.989(4)	15.5 ± 0.8	106.3 ± 5.2	41.2 ± 0.2

**Table 2:** Water adsorption data for Clean (CL) and Reduced (RD) spinel samples.

n		Heat of Adsorption, kJ/mol	Hydroxides, mol. %			H_2_O Coverage, Molecules/nm^2^
			Al–OH	Mg–OH	Total	
**Clean**	0.95	−75.1 ± 0.2	6.2 ± 0.3	13.7 ± 0.7	20.0 ± 1.0	12.2 ± 1.0
	1.07	−73.3 ± 0.4	34.0 ± 1.7	25.5 ± 1.3	59.4 ± 3.0	12.9 ± 0.1
	1.15	−71.0 ± 0.7	35.4 ± 1.8	12.4 ± 0.6	47.0 ± 2.4	11.5 ± 0.1
	2.45	−67.2 ± 0.2	32.1 ± 1.0	2.2 ± 0.1	34.3 ± 1.7	10.1 ± 0.1
**Reduced**	0.95	−71.0 ± 1.0	8.3 ± 0.4	16.9 ± 0.8	25.1 ± 1.2	15.3 ± 0.3
	1.07	−75.6 ± 0.9	30.3 ± 1.5	18.9 ± 0.9	48.2 ± 2.5	11.3 ± 0.2
	1.15	−68.5 ± 0.4	37.5 ± 1.9	11.5 ± 0.6	49.0 ± 2.4	13.4 ± 0.1
	2.45	−66.3 ± 0.3	41.3 ± 2.1	7.8 ± 0.4	49.0 ± 2.4	13.5 ± 0.2

**Table 3:** Water adsorption by MgO•2.45Al_2_O_3._

n		Inversion (i)	Heat of Adsorption, kJ/mol	Extent of Hydroxides Formed, mol. %			H_2_O Coverage, Molecules/nm^2^
				Al–OH	Mg–OH	Total	
**2.45**	Untreated	0.44	−67.2 ± 0.3	32.1 ± 1.6	2.2 ± 0.1	34.3 ± 1.7	10.1 ± 0.1
	Treated/(EF)	0.33	−62.5 ± 0.5	25.0 ± 1.3	1.8 ± 0.1	26.8 ± 1.4	8.8 ± 0.1

## Supplementary Materials

The following are available online at https://www.mdpi.com/1996-1944/13/14/3195/s1, Figure S1: Adsorption isotherms of the samples with clean surface, Figure S2: Adsorption isotherms of the samples with reduced surface, Table S1: Atomic percentage of Al and Mg oxides and hydroxides for adsorption processes with reduction, Table S2: Atomic percentage of Al and Mg oxides and hydroxides for adsorption processes with oxidation.
