# Supplementary material for: Effect of Structure and Composition of Non-Stoichiometry Magnesium Aluminate Spinel on Water Adsorption
Source: Materials (Basel). 2020 Jul 17;13(14):3195. doi: 10.3390/ma13143195 (PMC7412034; doi:10.3390/ma13143195)
Supplement: Supplementary file 1 [file materials-13-03195-s001.pdf]

materials

Supplementary Materials

Effect of Structure and Composition of Non-Stoichiometry Magnesium Aluminate Spinel on Water Adsorption

Yuval Mordekovitz, Yael Shoval, Natali Froumin, and Shmuel Hayun

This supplementary information includes the adsorption isotherm and the detailed XPS information.

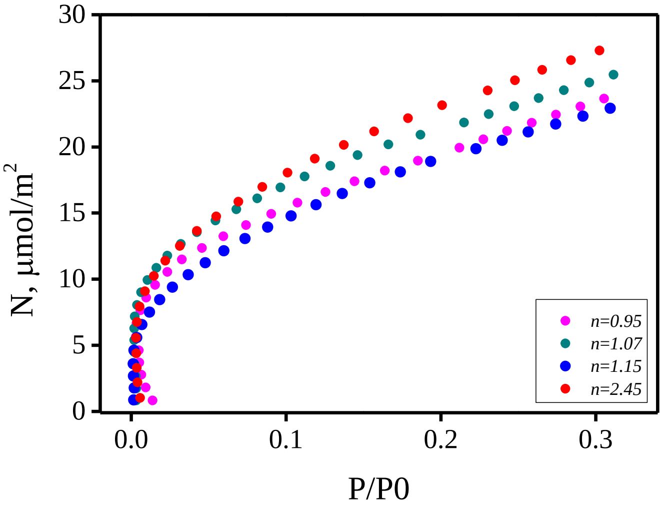

Figure S1. Adsorption isotherms of the samples with clean surface.

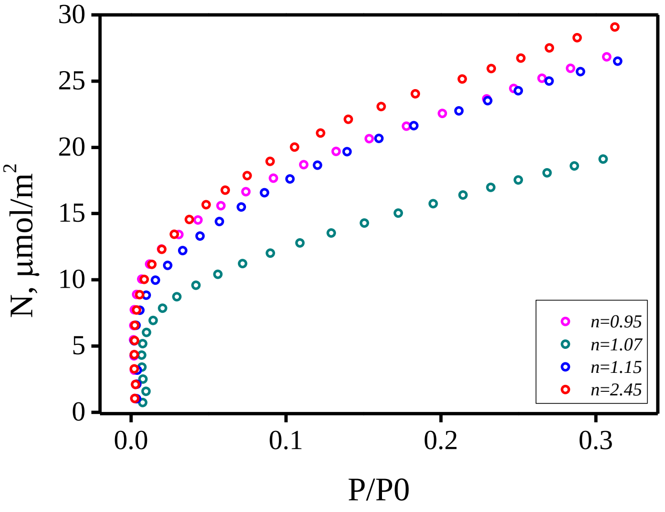

Figure S2. Adsorption isotherms of the samples with reduced surface.

materials

MDPI

Table S1. Atomic percentage of Al and Mg oxides and hydroxides for adsorption processes with reduction.

	**After Degas, at.%**				**After Water Adsorption, at.%**				**Formed Hydroxide, at.%**	
	**Al–O**	**Al–OH**	**Mg–O**	**Mg–OH**	**Al–O**	**Al–OH**	**Mg–O**	**Mg–OH**	**$Al(OH)_3$**	**$Mg(OH)_2$**
0.95	19.80 ± 0.99	13.08 ± 0.65	39.47 ± 1.97	28.03 ± 1.40	11.54 ± 0.57	21.34 ± 1.06	22.62 ± 1.13	44.47 ± 2.22	8.26 ± 0.41	16.85 ± 0.84
1.07	41.80 ± 2.09	11.39 ± 0.56	34.76 ± 1.73	11.98 ± 0.59	11.53 ± 0.57	39.05 ± 2.08	15.83 ± 0.79	30.91 ± 1.54	30.27 ± 1.51	18.93 ± 0.94
1.15	55.60 ± 2.78	13.71 ± 0.68	21.99 ± 1.09	8.70 ± 0.493	18.06 ± 0.90	51.24 ± 2.56	10.51 ± 0.52	20.18 ± 1.00	37.54 ± 1.87	11.48 ± 0.57
2.47	59.89 ± 2.99	21.88 ± 1.09	12.22 ± 0.61	5.94 ± 0.29	18.61 ± 0.93	63.20 ± 3.20	4.46 ± 0.22	13.53 ± 0.67	41.28 ± 2.06	7.76 ± 0.38

Table S2. Atomic percentage of Al and Mg oxides and hydroxides for adsorption processes with oxidation.

	**After Degas, at.%**				**After Water Adsorption, at.%**				**Formed Hydroxide, at.%**	
	**Al–O**	**Al–OH**	**Mg–O**	**Mg–OH**	**Al–O**	**Al–OH**	**Mg–O**	**Mg–OH**	**$Al(OH)_3$**	**$Mg(OH)_2$**
0.95	20.49 ± 1.02	12.39 ± 0.61	42.64 ± 2.13	24.46 ± 1.22	14.31 ± 0.71	18.57 ± 0.92	28.93 ± 1.44	38.17 ± 1.90	6.18 ± 0.31	13.71 ± 0.68
1.07	44.54 ± 2.22	8.70 ± 0.43	36.96 ± 1.84	9.78 ± 0.48	10.58 ± 0.52	42.66 ± 2.13	11.46 ± 0.57	35.28 ± 1.76	33.96 ± 1.70	25.50 ± 1.25
1.15	55.88 ± 2.79	13.43 ± 0.67	22.29 ± 1.11	8.40 ± 0.42	13.24 ± 0.66	56.06 ± 2.80	9.87 ± 0.49	20.82 ± 1.04	35.44 ± 1.77	12.42 ± 0.62
2.47	61.14 ± 3.05	20.67 ± 1.03	12.40 ± 0.62	5.77 ± 0.28	29.04 ± 1.45	52.77 ± 2.63	10.22 ± 0.51	7.92 ± 0.39	32.09 ± 1.60	2.18 ± 0.10

© 2020 by the authors. Submitted for possible open access publication under the terms and conditions of the Creative Commons Attribution (CC BY) license (http://creativecommons.org/licenses/by/4.0/).
